# Correlation of Longitudinal Gray Matter Volume Changes and Motor Recovery in Patients After Pontine Infarction

**DOI:** 10.3389/fneur.2018.00312

**Published:** 2018-06-01

**Authors:** Peipei Wang, Xiuqin Jia, Miao Zhang, Yanxiang Cao, Zhilian Zhao, Yi Shan, Qingfeng Ma, Tianyi Qian, Jingjuan Wang, Jie Lu, Kuncheng Li

**Affiliations:** ^1^Department of Radiology, Xuanwu Hospital, Capital Medical University, Beijing, China; ^2^Beijing Key Laboratory of Magnetic Resonance Imaging and Brain Informatics, Beijing, China; ^3^Institute of Psychology, Chinese Academy of Sciences, Beijing, China; ^4^Department of Radiology, Chaoyang Hospital, Capital Medical University, Beijing, China; ^5^Department of Neurology, Xuanwu Hospital, Capital Medical University, Beijing, China; ^6^Collaborations NE Asia, Siemens Healthcare, Beijing, China; ^7^Department of Nuclear Medicine, Xuanwu Hospital, Capital Medical University, Beijing, China

**Keywords:** pontine infarction, gray matter volume, voxel-based morphometry, magnetic resonance imaging, neuronal plasticity

## Abstract

The mechanisms of motor functional recovery after pontine infarction (PI) remain unclear. Here, we assessed longitudinal changes in gray matter volume (GMV) and examined the relationship between GMV and clinical outcome. Fifteen patients with unilateral PI underwent magnetic resonance imaging and neurological exams five times during a period of 6 months. Another 15 healthy participants were enrolled as the normal control (NC) group and were examined with the same protocol. The MR exam included routine protocol and a 3D T1-weighted magnetization-prepared rapid acquisition gradient echo scan. Changes in GMV were assessed using voxel-based morphometry. Furthermore, the correlations between GMV changes in regions of interest and clinical scores were assessed. Compared with NCs, the decreased GMVs in the contralateral uvula of cerebellum and the ipsilateral tuber of cerebellum were detected at third month after stroke onset. At the sixth month after stroke onset, the decreased GMVs were detected in the contralateral culmen of cerebellum, putamen, as well as in the ipsilateral tuber/tonsil of cerebellum. Compared with NC, the PI group exhibited significant increases in GMV at each follow-up time point relative to stroke onset. Specifically, the significant GMV increase was found in the ipsilateral middle frontal gyrus and ventral anterior nucleus of thalamus at second week after stroke onset. At first month after stroke onset, the increased GMVs in the ipsilateral middle temporal gyrus were detected. The significant GMV increase in the ipsilateral mediodorsal thalamus was noted at third month after stroke onset. At the end of sixth month after stroke onset, the GMV increase was found in the ipsilateral mediodorsal thalamus, superior frontal gyrus, and the contralateral precuneus. Across five times during a period of 6-month, a negative correlation was observed between mean GMV in the contralateral uvula, culmen, putamen, and ipsilateral tuber/tonsil and mean Fugl-Meyer (FM) score. However, mean GMV in the ipsilateral mediodorsal thalamus was positively correlated with mean FM score. Our findings suggest that structural reorganization of the ipsilateral mediodorsal thalamus might contribute to motor functional recovery after PI.

## Introduction

According to the World Health Organization, approximately 15 million people worldwide suffer stroke annually (1). Pontine infarction (PI) accounts for about 7% of all ischemic strokes (2), and approximately 15% of all posterior circulation cerebral infarctions are PIs (3). Although patients with PI consistently present with significant initial motor disability, most individuals show some improvement of motor function over the course of recovery (4, 5). However, the mechanisms underlying spontaneous recovery of motor function remain unclear.

Dynamic remodeling may lead to changes in brain structure, including atrophy and/or expansion of specific brain regions after ischemic stroke (5–7). One study reported that gray matter volume (GMV) was not correlated with motricity index scores, but that patients with decreased GMV exhibited a small degree of motor improvement after stroke (8). PI patients might experience dysfunction in motor areas that are remote from the location of the infarct. For example, Gauthier et al. found that the decreased gray matter (GM) density in noninfarcted motor regions was correlated with motor function and predicted poorer motor outcome and poorer rehabilitation therapy in patients with chronic stroke (9). Nonetheless, post-stroke increases in GMV in the contralateral hippocampus and precuneus were positively correlated with the recovery of motor function (8, 10). However, the relationship between structural changes and motor functional prognosis has not been completely elucidated.

One longitudinal study of PI patients showed progressive changes in fractional anisotropy (FA) values during a 6-month follow-up period using diffusion-tensor imaging. The FA values in the regions distal to the pontine infarct decreased within 1 week after onset, then gradually increased as the disease progressed above the pons. Compensatory changes in the corticospinal tract (CST) have been observed in some patients with severe CST damage during motor functional recovery (11). However, longitudinal GMV changes after PI have not been thoroughly investigated, and the mechanisms of motor functional recovery are still unclear. We hypothesized that isolated PI could lead to changes in the GM in remote areas of the brain and that changes in GMV might contribute to the restoration of function after stroke. In this study, we used voxel-based morphometry (VBM) to analyze GMV changes in PI patients from the acute to chronic stage and explored the relationship between GMV changes in specific brain regions and the recovery of motor function.

## Materials and Methods

### Participants

A total of 15 patients (4 females, 11 males; mean age: 58.1 years) with unilateral PI group (10 patients with PI in the right hemisphere) were recruited in this study. The criteria for enrollment were (1) the first episode of unilateral PI occurred within 7 days of symptom onset, as identified by magnetic resonance imaging (MRI); (2) no historical neurologic or psychiatric disorders; and (3) no other concomitant brain lesions or previous infarcts. The exclusion criteria were (1) multiple brain lesions; (2) unclear onset-time; (3) lesions outside the pons or extensive infarcts involving the midbrain or medulla; (4) recurrence of infarction or secondary hemorrhage during follow-up; and (5) deafness and/or blindness, or aphasia that might prevent completion of the study. As a normal control (NC) group, 15 healthy age-, sex-, and handedness-matched subjects with no history of psychiatric or neurological diseases were enrolled. Experiments were conducted with the understanding and written consent of each participant. The study procedures were approved by the Institutional Review Board of Xuanwu Hospital, Capital Medical University.

### Experimental Design

This longitudinal investigation consisted of neurological assessments and MRI exams conducted at five different time points. The PI patients were examined within 7 days, at 2 weeks, 1 month, 3 months, and 6 months after stroke onset. Those in the NC group were assessed five times within a 6-month period such that the time intervals between the assessments were consistent with those in the patient group. Two experienced neurologists conducted clinical neurological assessments of the patients using the Fugl-Meyer (FM) scale. The neurological function assessment was carried out on the same day as the MR examination.

### Image Acquisition

All subjects were examined using a Magnetom Trio Tim 3.0-T MR scanner (Siemens Healthcare, Erlangen, Germany) with a 12-channel head coil. Structural images were collected using a sagittal 3D-magnetization-prepared rapid acquisition gradient echo (MPRAGE) T1-weighted sequence with the following parameters: TR = 1,600 ms; TE = 2.15 ms; flip angle = 9°; FOV = 256 mm × 256 mm; matrix size = 256 × 256; voxel size = 1 mm × 1 mm × 1 mm. The 176 slices covered the entire brain, from the bottom of the cerebellum to the vertex and from ear to ear. Axial T1-weighted, fast spin-echo T2-weighted, fluid attenuation inversion recovery, and diffusion-weighted imaging (DWI) examinations were also performed.

### Behavioral Examination

At each visit, behavioral assessments were performed by two clinicians independently before and after the MRI examination. The two scores were averaged to provide a best estimate. Motor function, balance, sensation, and joint function of the upper limbs were assessed using 33 tasks. Each task was rated on a scale of 0–2 (0 indicates the subject was unable to perform the task, 1 indicates the subject could partially perform the task, and 2 indicates the subject was able to perform the task). The sum of the 33 scores was then normalized to a score between 0 and 100, where 100 represented good performance on all 33 tasks.

### Normalized Infarction Volume (NIV) Measurement

The infarction volume in each patient was measured using MRIcro software (version 1.40). Two experienced physicians individually measured the infarction size using the DWI images for patients in the sub-acute stage and the fluid attenuated inversion recovery images for chronic patients. The averages of the values produced by the two physicians were used to define the infarction volume in each patient. Given the individual differences in brain volume, the infarction volume of the subjects was normalized. The area on the middle sagittal plane of the brain is highly correlated with the volume of the brain (relation coefficient = 0.98) (12), and therefore, the median sagittal plane area was measured using the 3D-MPRAGE sequence to normalize the infarction size (13). The median sagittal area was identified automatically by the software. We used the following formula for normalization:

$$\text{normalized cerebral infarction volume}=\frac{\text{infarction volume}\times \text{average area of the median sagittal plane}}{\text{area of median sagittal plane of pontine patients}}.$$

### VBM-Diffeomorphic Anatomical Registration Through Exponentiated Lie Analysis

The MPRAGE images were processed using the VBM toolbox in the SPM8 software package (Statistical Parametric Mapping, Wellcome Department of Imaging Neuroscience, London, UK), and the Diffeomorphic Anatomical Registration Through Exponentiated Lie algebra algorithm was applied for registration (14). First, the MPRAGE data from the patients with lesions in the left hemisphere were flipped from left to right (15). Then, we defined the right cerebral hemisphere as the ipsilateral side, and the left hemisphere as the contralateral side. Pre-processing for the VBM method included the following steps. (1) A standard segmentation algorithm in SPM8 was used to segment MPR images into GM, white matter, and cerebrospinal fluid. (2) To obtain a specific GM template for the PI and NC groups, a Diffeomorphic Anatomical Registration Through Exponentiated Lie method was employed to normalize the GM images from the two groups. (3) We normalized the GMVs of the two groups to the GM templates in Montreal Neurological Institute (MNI) space. Jacobin determinant was used to calculate the size of the deformation area and generate information on the volume change for each individual at each time point. Next, the nonlinear deformation method was used to standardize the gray images into MNI space and to generate volume modulation. (4) The normalized GM images (1.5 mm × 1.5 mm × 1.5 mm) were smoothed using a three-dimensional Gaussian kernel (full width at half maximum was 6 mm). (5) Then, the sample homogeneity of the normalized data was checked for the NC group.

### Statistical Analysis

To analyze the demographic data, the two-related samples *t*-test and the Chi-square test were used for dichotomous variables. One-way analysis of variance (ANOVA) was used to compare the differences in NIV and FM scores at different time points in the PI group, and *post hoc* analyses were performed to identify differences between time points. The statistical significance threshold was set *p* < 0.05. The Pearson correlation analysis was performed to evaluate the relationship between the NIV and FM scores. All statistical analyses were performed using SPSS, version 17.

For the GMV changes, the present study was organized into two (group: PI versus NC) × 5 (time: within 7 days, 2 weeks, 1 month, 3 months, and 6 months after stroke onset) flexible factorial designs. Thus, we had 10 conditions during a period of 6 months for two groups: PI within 7 days (PI1), PI at 2 weeks (PI2), PI at 1 month (PI3), PI at 3 months (PI4), PI at 6 months (PI5), NC at baseline (NC1), NC at 2 weeks (NC2), NC at 1 month (NC3), NC at 3 months (NC4), and NC at 6 months (NC5). Gender, age, total intracranial volume (TIV), and NIV (0 for NCs) were entered as nuisance variables into the flexible factorial design.

The interaction effects of “group” by “time” were revealed by the following eight contrasts: the decreased GM volumes specific to PI patients were assessed at five time points by calculating the contrast of [(PI1–PI2) − (NC1–NC2)], [(PI1–PI3) − (NC1–NC3)], [(PI1–PI4) − (NC1–NC4)], and [(PI1–PI5) − (NC1–NC5)], and increased GM volumes specific to PI patients at different time points were evaluated by calculating the contrast of [(PI2–PI1) − (NC2–NC1)], [(PI3–PI1) − (NC3–NC1)], [(PI4–PI1) − (NC4–NC1)], and [(PI5–PI1) − (NC5–NC1)]. The statistical threshold was set at *p* < 0.001 (uncorrected; cluster size ≥ 100 voxels) for detecting GMV changes during a 6-month period.

### Regions of Interest (ROIs) Analysis

To further investigate the relationship between changes in FM scores and GMV in specific brain regions, we defined ROIs using a cluster-based method. On the basis of previous longitudinal or cross-sectional studies, GMV changes in the brain regions of cerebellum anterior and posterior lobe, putamen, premotor motor cortex/supplementary motor cortex, and thalamus were associated with stroke patients (5, 6, 16). The following clusters were defined as ROIs: contralateral uvula of the cerebellum, 201 voxels; MNI coordinates: −36, −78, −36; contralateral culmen of the cerebellum, 496 voxels; MNI coordinates: −12, −56, −21; contralateral putamen, 132 voxels; MNI coordinates: −23, 3, 8; ipsilateral ventral anterior nucleus of the thalamus, 153 voxels; MNI coordinates: 6, −5, 8; and ipsilateral middle frontal gyrus, 166 voxels; MNI coordinates: 35, −11, 68. For the interested regions appeared at different times, the conjunction analysis was conducted and the resultant area defined as ROIs. The additional clusters were defined as ROIs by conjunction: ipsilateral tuber/tonsil of the cerebellum, 102 voxels; MNI coordinates: 32, −47, −41 and ipsilateral mediodorsal thalamus, 184 voxels; MNI coordinates: 8, −23, 14. The GMV values in these ROIs were extracted. Results were localized by xjView software (http://www.alivelearn.net/xjview).

### Correlation Analysis

To further explore the temporal dynamic relationship between the GMV changes in ROIs and the motor function recovery measured by FM score, Pearson correlation analysis was performed to determine any potential association between mean GMV and mean FM scores across five times during a period of 6-month. The statistical threshold was set at *p* < 0.05 (uncorrected).

## Results

### Demographic and Clinical Findings

**Figure 1:**
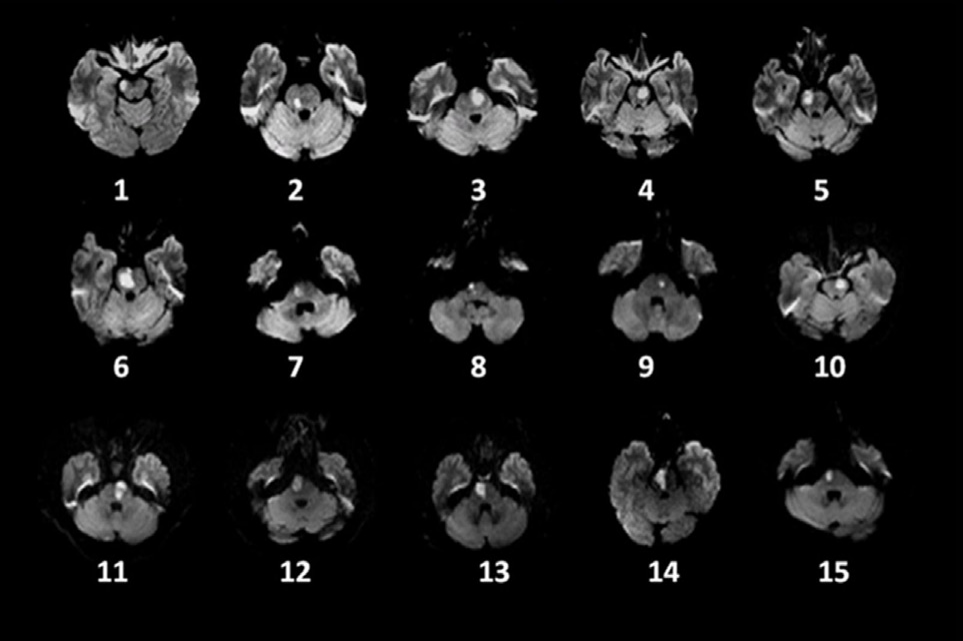
Distribution of lesions on diffusion-weighted imaging in 15 patients with pontine infarction.

Demographic information and clinical findings for the 15 PI patients at the five different assessment times are listed in Table 1. Lesion locations are shown on axial slices of the diffusion-weighted images, taken within the seventh day after stroke onset (Figure 1). There were no significant sex and age differences between the PI and control patients (*p* > 0.05).

**Table 1:** Demographic features and clinical data of the PI group.

Patient no.	1	2	3	4	5	6	7	8	9	10	11	12	13	14	15
Age (years)	54	57	59	48	63	60	61	68	62	62	60	56	64	42	56
Gender	F	M	M	M	M	M	M	M	M	M	F	M	F	M	F
FM1	75	88.6	66.7	50	62.9	42.4	75	100	72.7	45.5	0	27.3	6.1	15.2	0
FM2	90.9	94.7	81.8	68.2	83.3	59.9	90.9	100	90.2	72.7	34.9	47	7.6	28.8	50
FM3	95.5	96.2	86.4	75.8	96.2	84.6	99.2	100	98.5	81.1	69.7	89.4	40.9	46.8	80.3
FM4	98.5	100	100	74.3	100	98.5	98.5	100	90.9	77.3	83.3	98.5	60.6	69.7	90.9
FM5	100	99.2	97	78	100	100	97	100	98.5	85.6	95.5	100	74.2	83.3	100
NIV(ml)1	6.2	6.3	14.8	13	10	24	5.1	2.4	4.9	20	21.8	15.3	10.9	23.2	5.3
NIV(ml)2	3.8	4.1	16.2	9.9	7.6	20	7	1.3	2.6	18	18.7	7.8	7.4	15.8	2.4
NIV(ml)3	3.4	3.4	13.2	3.93	3.6	10.7	1.5	1.3	1.6	14.4	11.5	3.2	5.4	7.6	1.6
NIV(ml)4	1.9	3.1	9.9	4.8	3.1	10.2	3.3	0.8	0.4	9.6	9.6	4.7	5.5	9.7	2.8
NIV(ml)5	1.5	3.0	9.4	3.7	2.5	6.8	1.8	0.7	0.3	12.2	13.7	5	3.5	11.6	2.2
TIV(ml)1	1,473	1,466	1,479	1,549	1,702	1,530	1,621	1,576	1,547	1,318	1,294	1,711	1,297	1,760	1,532
TIV(ml)2	1,470	1,453	1,475	1,554	1,699	1,536	1,617	1,564	1,550	1,309	1,280	1,705	1,356	1,750	1,530
TIV(ml)3	1,467	1,454	1,479	1,550	1,690	1,522	1,620	1,574	1,539	1,312	1,282	1,704	1,538	1,757	1,521
TIV(ml)4	1,471	1,396	1,437	1,550	1,680	1,554	1,611	1,572	1,543	1,292	1,266	1,726	1,302	1,770	1,526
TIV(ml)5	1,478	1,453	1,439	1,543	1,693	1,545	1,603	1,575	1,544	1,287	1,284	1,647	1,370	1,766	1,533
Lesion side	R	R	L	L	R	R	R	R	L	L	L	R	R	R	R

*FM 1–5 represent the specific FM for each patient at each time point, NIV 1–5 represent the specific NIV for each patient at each time point, and TIV 1–5 represent the specific TIV for each patient at each time point*.

*F, female; M, male; FM, Fugl-Meyer (0–100); NIV, normalized infarction volume; TIV, total intracranial volume; R, right; L, left; PI, pontine infarction*.

### The NIVs and FM Scores in the PI Group

One-way ANOVA revealed that NIV decreased significantly during the observation period (*F* = 5.000, *p* = 0.001). The NIVs at each time point (PI1−5, respectively) were 12.21 ± 1.90, 9.50 ± 1.69, 5.75 ± 1.17, 5.29 ± 0.92, and 5.19 ± 1.15 ml. *Post hoc* analysis showed that the infarction volumes were significantly different between the seventh day and 1, 3, and 6 months after stroke onset (*p* < 0.05), as well as between 2 weeks and 3 and 6 months after stroke onset (*p* < 0.05).

The FM scores of all patients increased with time after stroke onset. FM scores changed significantly over time (*F* = 10.739, *p* = 0.000). FM scores on the seventh day after stroke onset were significantly different compared with 2 weeks and 1, 3, and 6 months after stroke onset (*p* < 0.05). FM scores at 2 weeks after stroke onset were significantly different from 3 and 6 months after stroke onset (*p* < 0.05). No significant correlations were observed between NIV obtained at the first time point and FM scores at each time point (<7 days, 2 weeks, 1 month, 3 months, 6 months after stroke onset) (corrected, *p* > 0.05).

### Whole-Brain GMV Changes

The significant changes in GMV in the various brain areas at different time points in the PI group were shown in Table 2. Compared with NC, the PI group exhibited significant decreases in GMV at third month (PI4) and sixth month (PI5) relative to stroke onset. The decreased GMVs in the contralateral uvula of cerebellum and the ipsilateral tuber of cerebellum were detected at third month after stroke onset. At the sixth month after stroke onset, the decreased GMVs were detected in the contralateral culmen of cerebellum, putamen, as well as in the ipsilateral cerebellar tuber/tonsil of cerebellum. Compared with NC, the PI group exhibited significant increases in GMV at each follow-up time point relative to stroke onset. Specifically, the significant GMV increase was found in the ipsilateral middle frontal gyrus and ventral anterior nucleus of thalamus at second week (PI2) after stroke onset. At first month (PI3) after stroke onset, the increased GMV in the ipsilateral middle temporal gyrus were detected. The significant GMV increase in the ipsilateral mediodorsal thalamus was noted at third month (PI4) after stroke onset. At the end of sixth month (PI5) after stroke onset, the GMV increase was found in the ipsilateral mediodorsal thalamus, superior frontal gyrus, and the contralateral precuneus.

**Table 2:** Whole-brain GMV changes in PI group compared with NC group.

Brain regions		MNI			
	Cluster size (voxels)	*x*	*y*	*z*	*T*-value
**Decreased GMV**					
(PI1–PI4) − (NC1–NC4)					
IL. CPLT	143	41	−71	−35	3.50
CL. CPLU	201	−36	−78	−36	3.55
(PI1–PI5) − (NC1–NC5)					
IL. CPLT/CPLCT	383	41	−65	−36	3.74
CL. CALC	496	−12	−56	−21	4.33
CL. Putamen	132	−23	3	8	3.60
**Increased GMV**					
(PI2–PI1) − (NC2–NC1)					
IL. Thalamus (VA)	153	6	−5	8	3.94
IL. MFG	166	35	−11	68	3.87
(PI3–PI1) − (NC3–NC1)					
IL. MTG	431	56	−72	20	4.56
(PI4–PI1) − (NC4–NC1)					
IL. Thalamus (MD)	610	8	−23	12	3.90
(PI5–PI1) − (NC5–NC1)					
IL. Thalamus (MD)	187	8	−23	14	3.78
IL. SFG	376	29	47	35	4.37
CL. Precuneus	164	−12	−83	47	3.76

*CPLT, cerebellum posterior lobe tuber; CPLU, cerebellum posterior lobe uvula; CPLCT, cerebellum posterior lobe cerebellar tonsil; CALC, cerebellum anterior lobe culmen; MFG, middle frontal gyrus; VA, ventral anterior; MTG, middle temporal gyrus; MD, mediodorsal; SFG, superior frontal gyrus; IL, ipsilateral; CL, contralateral; MNI, Montreal Neurological Institute; GMV, gray matter volume; NC, normal control*.

### Correlation Results

**Figure 2:**
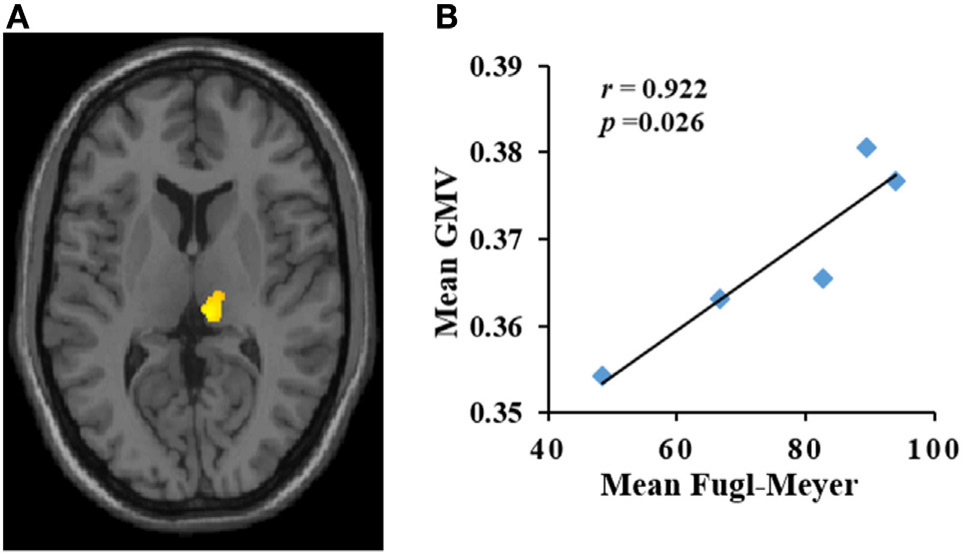
**(A,B)** Increase in gray matter volume (GMV) (*y*-axis) in the ipsilateral mediodorsal thalamus was positively correlated with Fugl-Meyer (*x*-axis) score in patients with pontine infarction during the period of observation. *r* indicates Pearson correlation coefficient.

**Figure 3:**
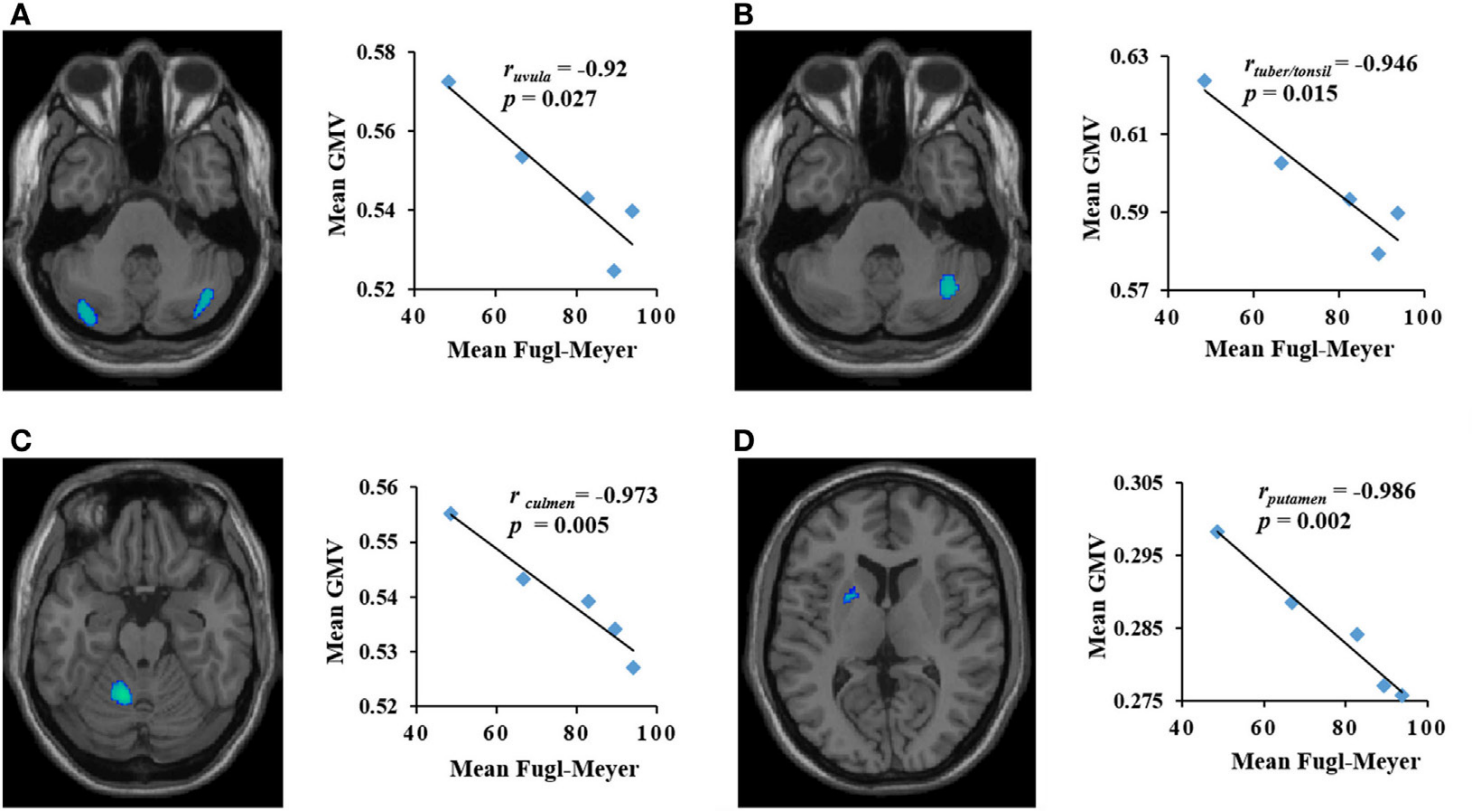
The brain areas with decreased gray matter volume (GMV) (*y*-axis) were negatively correlated with Fugl-Meyer score (*x*-axis) in patients with pontine infarction during the period of observation. *r* indicates Pearson correlation coefficient. **(A)** Contralateral uvula: *r* = −0.92, *p* = 0.027; **(B)** ipsilateral tuber/tonsil: *r* = −0.946, *p* = 0.015; **(C)** contralateral culmen: *r* = −0.973, *p* = 0.005; **(D)** contralateral putamen: *r* = −0.986, *p* = 0.002.

Dynamic changes of GMV values of ROIs in the contralateral uvula, culmen, putamen, ipsilateral tuber/tonsil, and mediodorsal thalamus were significantly correlated with changes in FM scores in the PI patients during the period of observation. The mean GMV in the ipsilateral mediodorsal thalamus was positively correlated with mean FM score (*r* = 0.922, *p* = 0.026) (Figure 2). The mean GMV in the contralateral uvula, culmen, putamen, and ipsilateral tuber/tonsil were negatively correlated with mean FM score [*r*_uvula_ = −0.92, *p* = 0.027 (Figure 3A); *r*_tuber/tonsil_ = −0.946, *p* = 0.015 (Figure 3B); *r*_culmen_ = −0.973, *p* = 0.005 (Figure 3C); *r*_putamen_ = −0.986, *p* = 0.002 (Figure 3D)].

## Discussion

In the present study, we investigated the relationship between changes in GMV and motor functional reorganization in individuals with unilateral pontine infraction. We evaluated participants over a period of approximately 6 months from the baseline to the follow-up assessments. Results showed some regions with significant change were distant from the primary lesion site in PI patients. Across five times during a period of 6-month, the increases in GMV in the ipsilateral mediodorsal thalamus were positively correlated with the recovery of motor function, while, a negative correlation was found between FM scores and GMV in the contralateral uvula, culmen, putamen, and ipsilateral tuber/tonsil. This suggests that the spontaneous recovery of motor function involves regional structural plasticity in PI patients.

We found that increased GMVs were primarily located in the ipsilateral hemisphere, which is consistent with previous studies suggesting that ipsilateral hemispheric reorganization contributes to the recovery of motor function in stroke patients. Indeed, animal studies have also found reorganization in the ipsilateral cortex after small lesions (17). In addition, many animal and human studies have shown that the contralateral cortex plays a minor role in recovery after stroke; instead, the plastic response comes mainly from the ipsilateral cortex (18–20). These observations are in line with functional MRI (fMRI) studies. A number of serial fMRI studies demonstrate a shift in the laterality of activation after stroke. Furthermore, studies have shown that brain activations are mainly located in the contralateral sensorimotor cortex when the affected paw is stimulated in animals. Later after stroke, activity shifts toward the ipsilateral sensorimotor cortex (21, 22). In patients with stroke, the volume of sensorimotor cortex activation in the ipsilateral hemisphere is associated with functional recovery (23). One previous study reported that neonatal primates subjected to unilateral lesion of the forelimb motor cortical region fully recovered hand function by adulthood. Inactivation of the contralateral M1 had no effect on the function of the unaffected hand, whereas inactivation of the ipsilateral M1 affected the functioning of the dysfunctional hand (20). This suggests that control of the affected extremity had shifted to a region of the cortex adjacent to the lesion. Such functional recovery occurred spontaneously after stroke, and preclinical studies have reported that peri-infarct regions, whether close or distant to the lesion, “take over” control of the disrupted functions (24, 25).

Interestingly, we observed that the increased GMV in the ipsilateral mediodorsal thalamus was positively correlated with the progressive gains in motor function. Thus, the structural plasticity of this brain area may be involved in the spontaneous recovery of motor function in PI patients. We mainly focus on the dynamic GMV changes in this region over time and explored the possible mechanism for reinstatement of the mediodorsal thalamus. We found a trend of initial GMV reduction in the ipsilateral mediodorsal thalamus of PI patients from seventh day to first month after stroke onset, then gradually increased to the normal level at follow-up time of third and sixth month after stroke onset relative to NC. Compared with NC group, a significant difference of GMV in the ipsilateral mediodorsal thalamus at the first time point of following up. The most possible explanation for this finding is reduction of GMV correlated with cerebrovascular disease risk factors in PI group. Hypertension (high blood pressure) is the important contributing risk factor for stroke, and it is widely acknowledged that hypertension is associated with shrinking in whole or local brain volume (26). These authors found brain atrophy of deep gray structure (for example, thalamus) could be detected in hypertension patients and those caused by projection fibers interrupted (27). Of course, we expected future work (collecting the risk factors of stroke both two groups) can be performed to strengthen this view.

However, comparing the PI group, the GMV gradually increased in the ipsilateral mediodorsal thalamus from second week to sixth month after stroke onset, and those was significantly different between seventh day, third and sixth month after stroke onset. The further result showed the increased GMV in thalamus correlated with the restoration of motor function at first, third, and sixth month after stroke onset. We demonstrated that the increased GMV in ipsilateral mediodorsal thalamus was associated with progressive gains in motor function. The thalamus is an important GM region that functions as a relay station, connecting different subcortical areas to the cerebral cortex (28). In particular, the mediodorsal thalamic nucleus, which is connected to sub-hippocampal structures, appears to play a critical role in memory (29–31). Previous studies have suggested that the structural plasticity of cognition-related areas (i.e., the hippocampus and precuneus) is facilitated concomitant with the recovery of motor function after subcortical stroke (8). In addition, Gauthier et al. found that structural reorganization in cognition-related brain areas contributes to the recovery of motor function in stroke patients after constraint-induced movement therapy (10). Indeed, it is well established that remodeling of cognition-related brain regions is important for the recovery of motor function after infarction (32). Therefore, as PI patients with significant increases in GMV in ipsilateral cognition-related regions experienced a higher degree of improvement in motor function, memory strategy-based interventions might be helpful in improving motor functional prognosis.

In this study, increases in GMV were observed in the PI group in parallel with decreases in areas distant from the primary lesion site. Three to six months after onset, the areas that showed decreases were almost all located in the cerebellum. Previous studies reported structural damage in brain areas that were both directly and indirectly connected to infarct lesions. The regions in which we found GMV reductions in the cerebellum were similar to those in a previous report that identified decreased GMV in the contralateral cerebellum after subcortical stroke (8), although a 12-week longitudinal study found no GMV decrease in the cerebellum after subcortical stroke (16). The discrepancy between our results and those reported by Fan et al. and Dang et al. might be caused by variation in the location of the infarction. Subcortical infarctions (motor cortex or internal capsule) mainly affect the CSTs (33). However, the ipsilateral tract and contralateral pontocerebellar fibers can be directly damaged by ischemic events in the pontine (34). Pontocerebellar fibers connect the cerebellar cortex and contralateral pontine nuclei, crossing the midline at an upper pontine level. Reductions in the bilateral cerebella are known to result from anterograde transsynaptic degeneration of pontocerebellar fibers. However, the mechanisms underlying structural atrophy after cerebral infarction are not fully understood. Axonal degeneration, myelin collapse, chronic insufficiency of regional cerebral blood flow, and compromised metabolism may contribute to the decrease in brain volume in stroke patients, while retrograde degeneration or anterograde transsynaptic degeneration is the most important cause of brain atrophy in cerebrovascular diseases (35, 36). Secondary degeneration caused by focal motor pathway stroke has also been widely reported in previous studies using diffusion tensor imaging, and the correlation between degeneration in the cerebral area and functional outcome has been established in patients with chronic stage stroke (37). We found a significant negative correlation between FM scores and GMV in the contralateral uvula, culmen, putamen, and ipsilateral tuber/tonsil. The more pronounced the degeneration, the more severe the motor deficit (38). This finding indicates that secondary degeneration in these areas might hamper functional recovery in patients with pontine infarct.

During the 6-month period after stroke, decreases and increases in the GMV were found in brain areas that were remote from the primary lesion. It has been showed that the structural changes after PI affect the motor recovery. In addition, increases in GMV in the ipsilateral mediodorsal thalamus were positively correlated with improved motor function, indicating that structural plasticity in cognition-related areas may contribute to the recovery of motor function in pontine infarct patients. However, decreased GMVs in the contralateral uvula, culmen, putamen, and ipsilateral tuber/tonsil, which were negatively correlated with FM score, may inhibit motor functional recovery. Our findings have important clinical prognostic implications for guiding rehabilitation therapy for pontine infarct patients.

There are several limitations in our study. First, the relatively small sample size may prevent us to provide conclusive of evidence for GMV changes in the brain after PI. We expected to expand sample size in our future work and our findings could be strengthened. Second, the cognitive function was not assessed in our study, which may affect our GMV analysis. Future studies must address the cognitive function changes in patients with PI.

## Ethics Statement

The study protocol was approved by the Ethics Committee of Xuanwu Hospital of Capital Medical University, Beijing, China. Written informed consent in accordance with the Declaration of Helsinki was obtained from each participant of this study.

## Author Contributions

PW: volunteer recruitment, experimental design, data collection, statistics, and manuscript preparation. XJ: data analysis and statistics. MZ, YC, ZZ, and YS: patient recruitment and data collection. QM: patient recruitment and clinical data collection. TQ: advice regarding data analysis, scanning parameters design, and manuscript preparation. JW: manuscript submission. JL: conception, funding, study design, supervision, and manuscript preparation. KL: funding, supervision, and manuscript preparation.

## Conflict of Interest Statement

TQ was employed by company Siemens Healthcare (China). The remaining authors declare that the research was conducted in the absence of any commercial or financial relationships that could be construed as a potential conflict of interest.

## Abbreviations

GMV, gray matter volume; MRI, magnetic resonance imaging; VBM, voxel-based morphometry; NC, normal control; FM, Fugl-Meyer; PI, pontine infarction; MI, motricity index; FA, fractional anisotropy; CST, corticospinal tract; MPRAGE, magnetization-prepared rapid acquisition gradient echo; DWI, diffusion-weighted imaging; FLAIR, fluid attenuated inversion recovery; GW, gray matter; WM, white matter; CSF, cerebrospinal fluid; ANOVA, analysis of variance; TIV, total intracranial volume; ROIs, regions of interest; NIV, normalized infarction volume; fMRI, functional MRI.
