# Supplementary figures and images for: Correlation of Longitudinal Gray Matter Volume Changes and Motor Recovery in Patients After Pontine Infarction
Source: Front Neurol. 2018 Jun 1;9:312. doi: 10.3389/fneur.2018.00312 (PMC5992285; doi:10.3389/fneur.2018.00312)

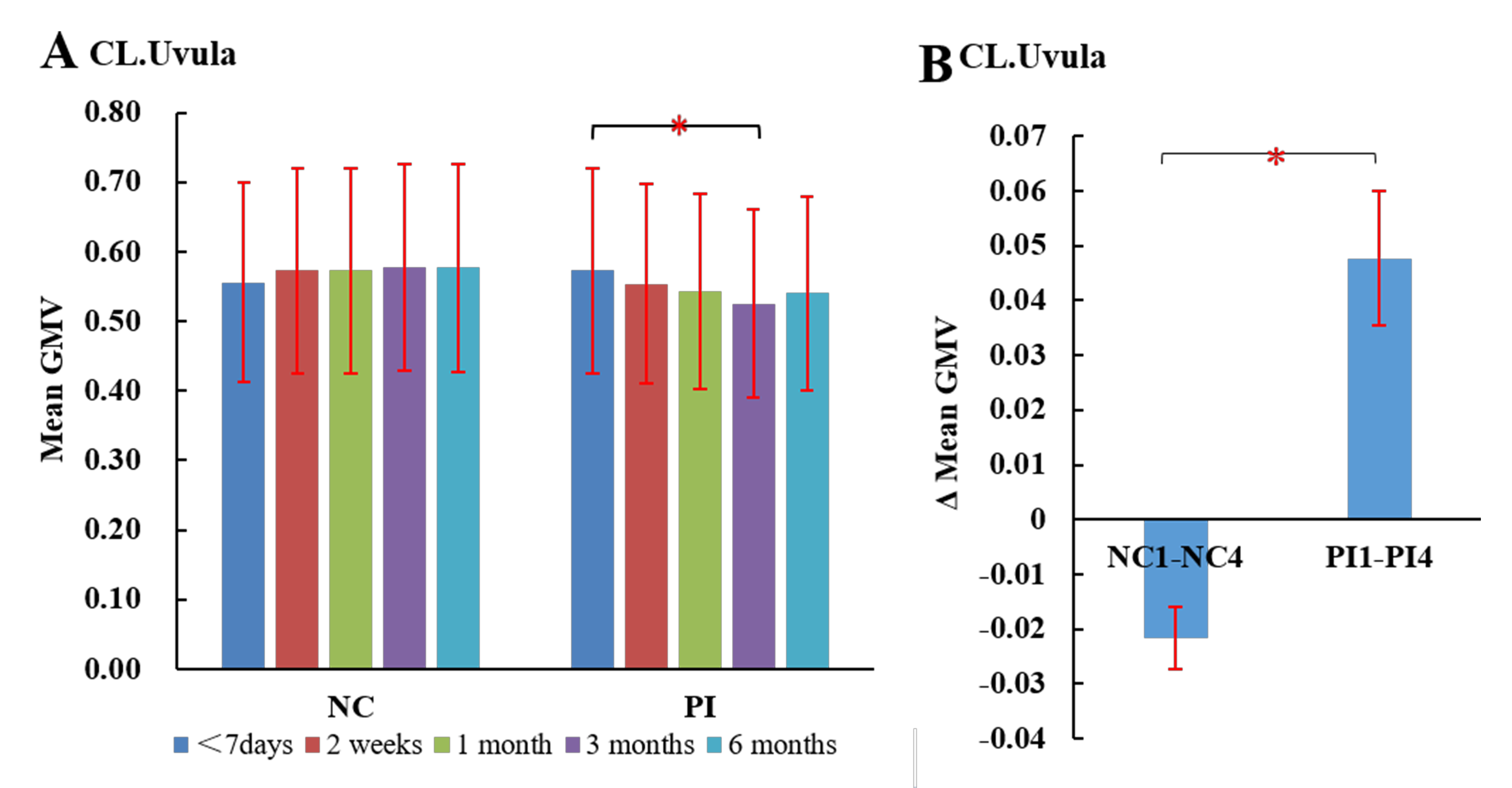

Supplement: Supplementary file 1 [file Image_1.tif]

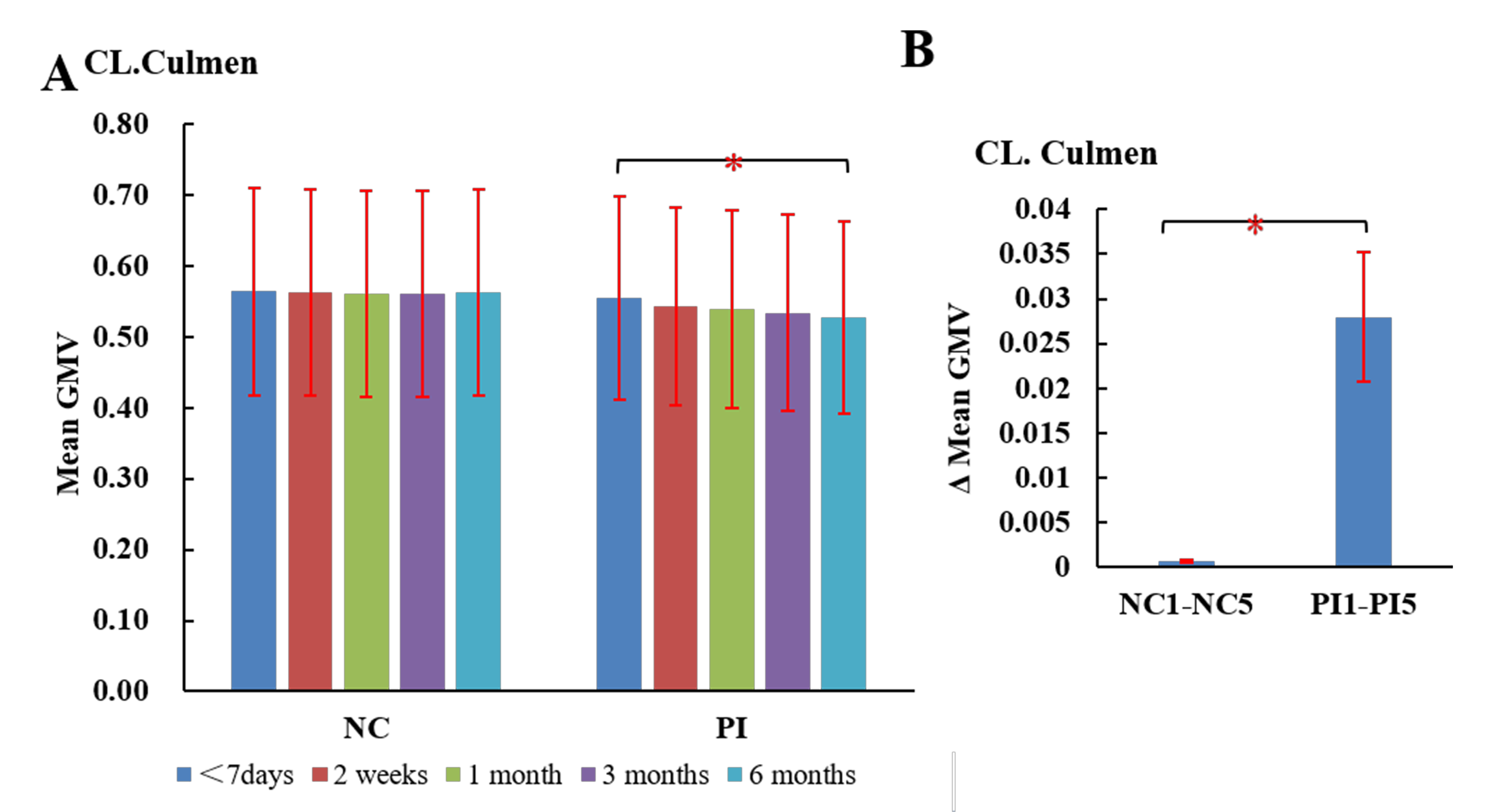

Supplement: Supplementary file 2 [file Image_2.tif]

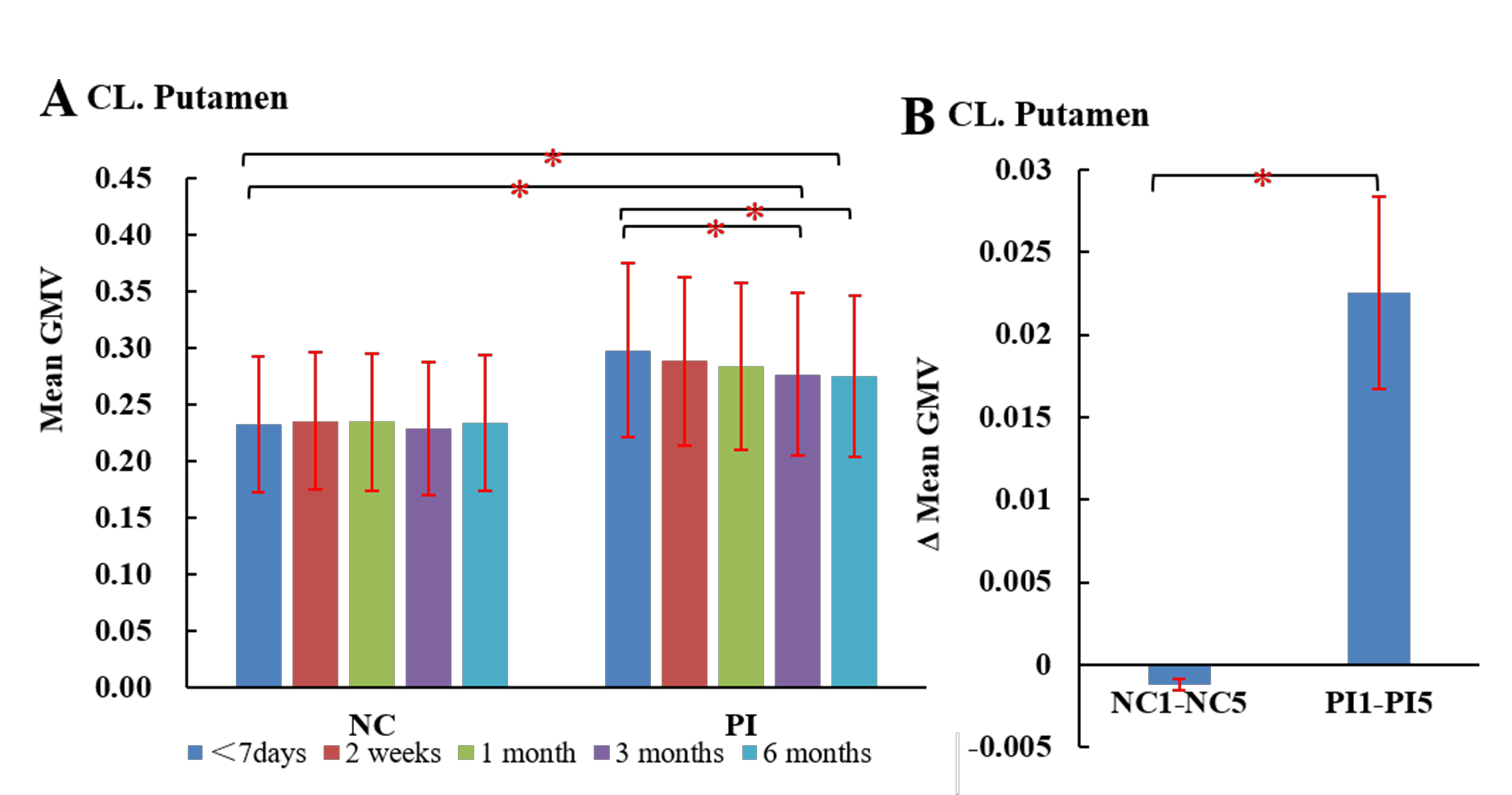

Supplement: Supplementary file 3 [file Image_3.tif]

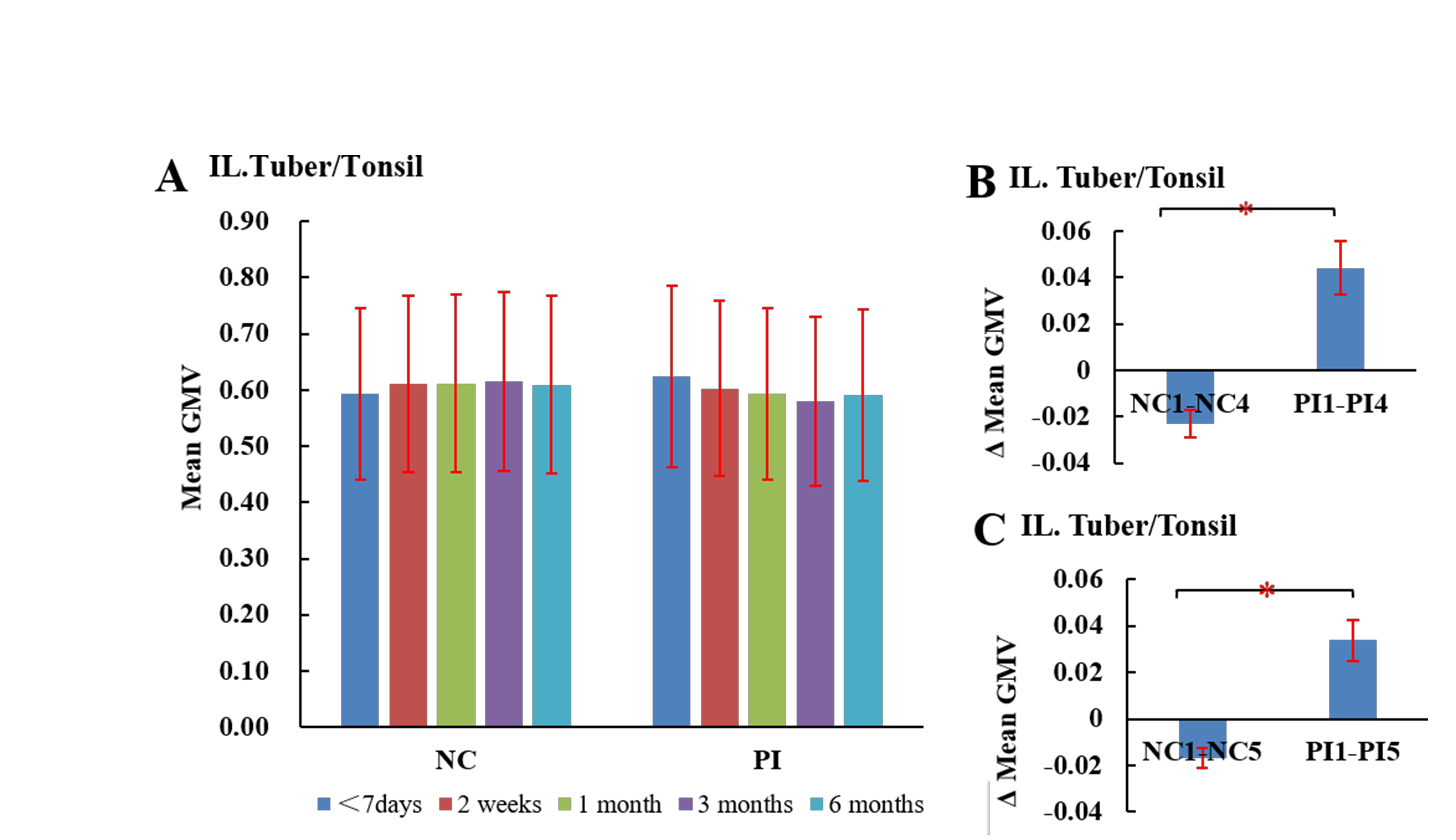

Supplement: Supplementary file 4 [file Image_4.tif]

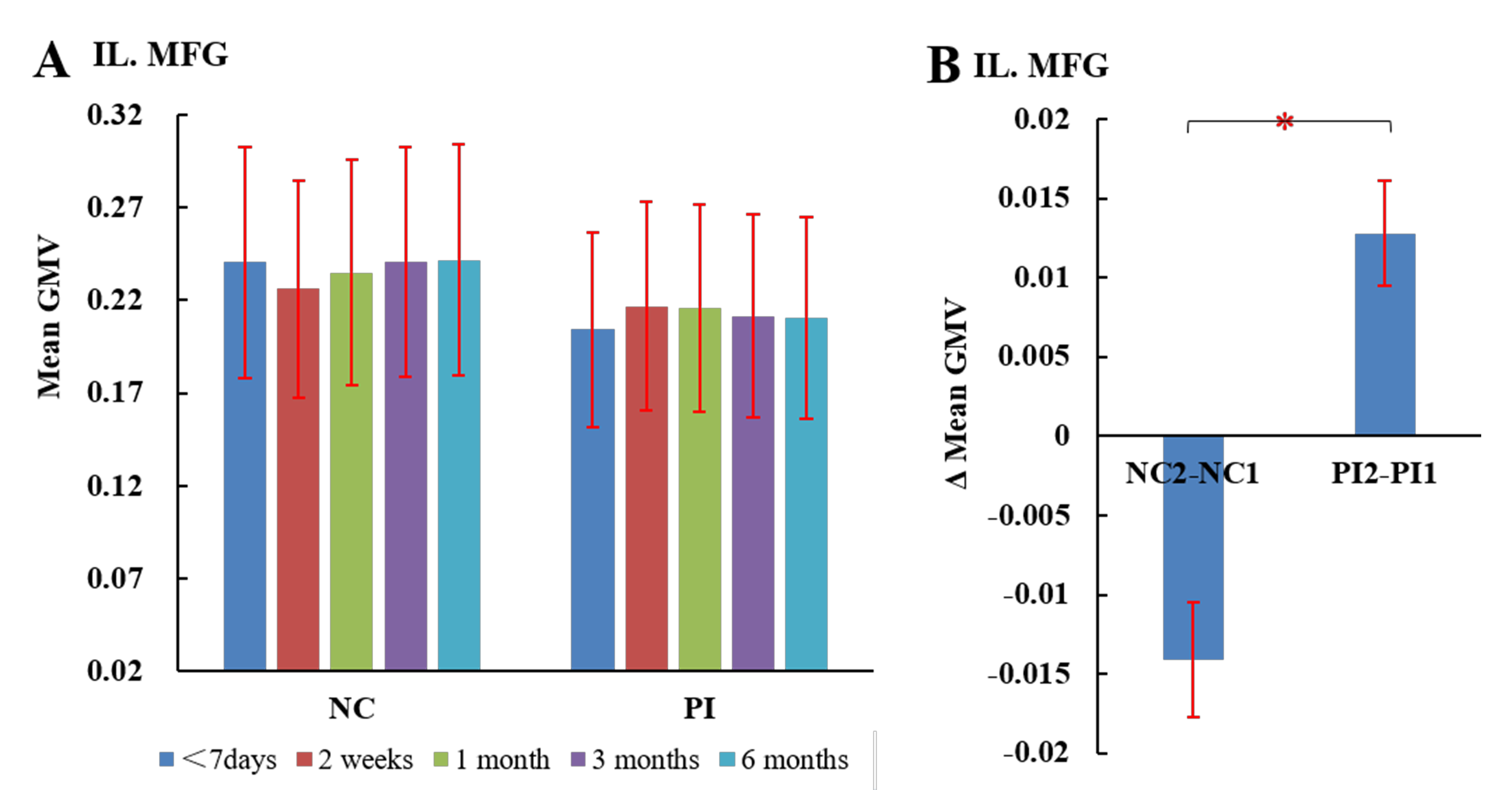

Supplement: Supplementary file 5 [file Image_5.tif]

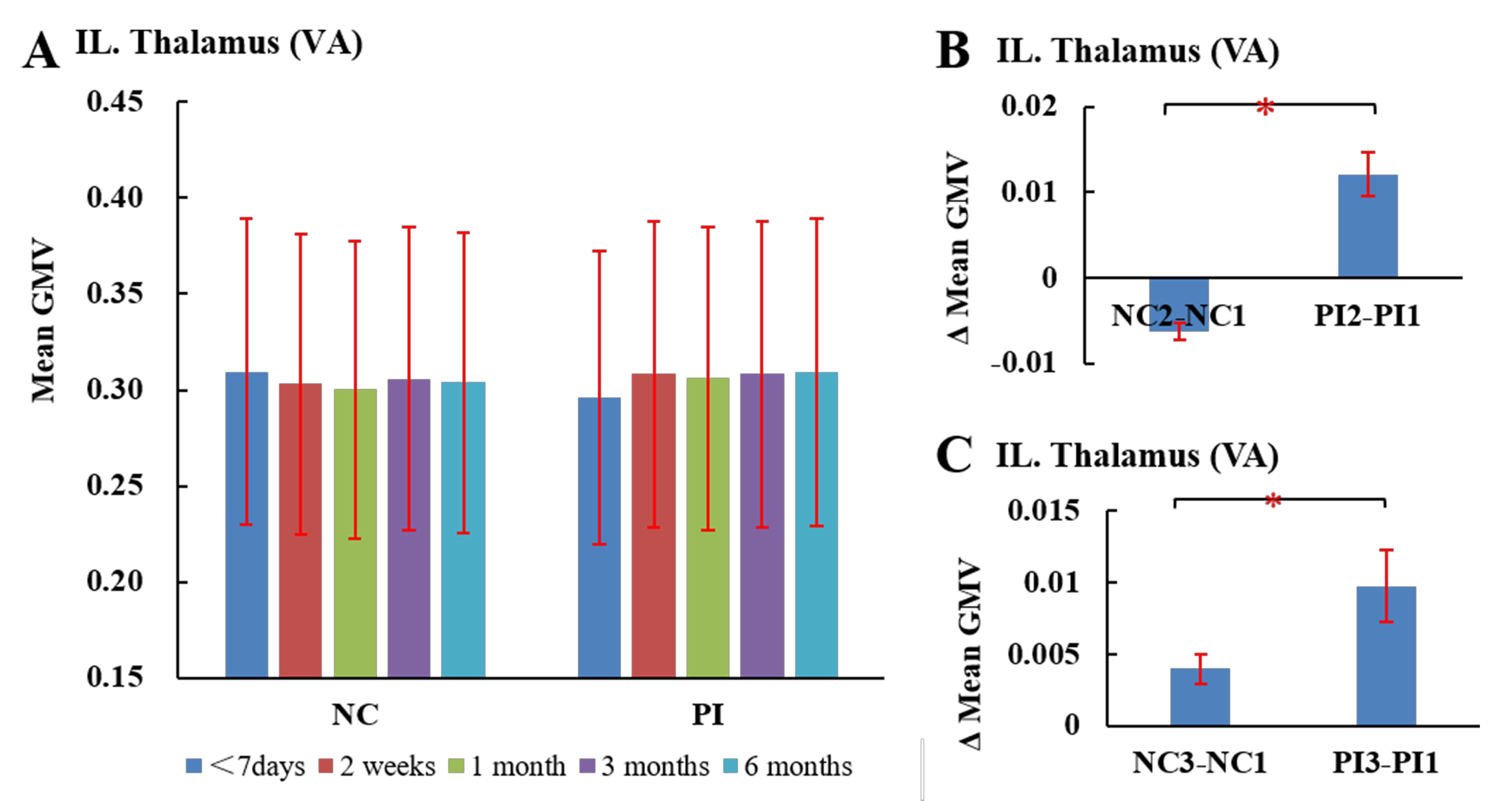

Supplement: Supplementary file 6 [file Image_6.tif]

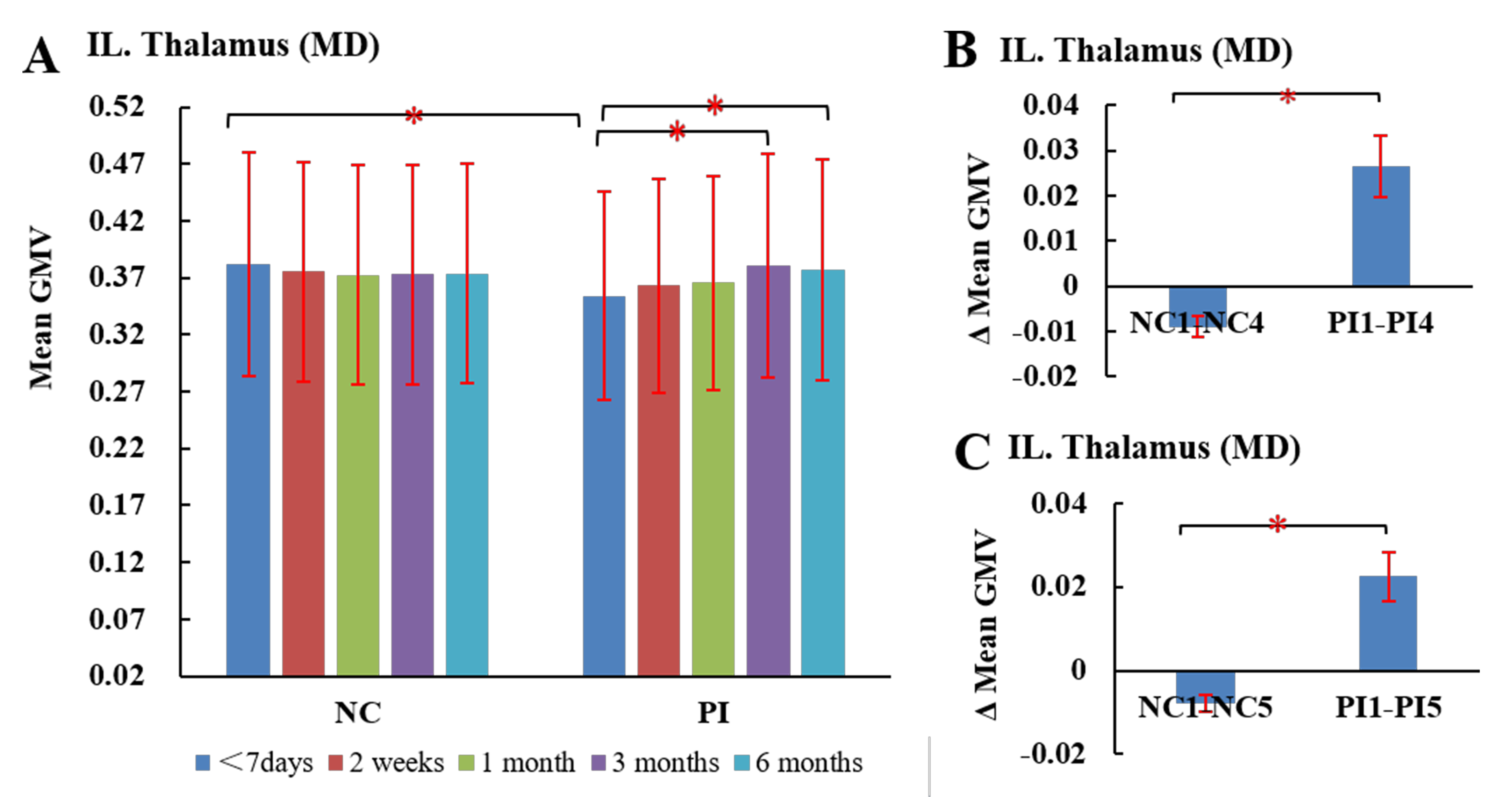

Supplement: Supplementary file 7 [file Image_7.tif]
