# Supplementary material for: Correlation of Longitudinal Gray Matter Volume Changes and Motor Recovery in Patients After Pontine Infarction
Source: Front Neurol. 2018 Jun 1;9:312. doi: 10.3389/fneur.2018.00312 (PMC5992285; doi:10.3389/fneur.2018.00312)
Supplement: Supplementary file 8 [file Data_Sheet_1.docx]

**TABLE 1 | NIHSS of the PI group**

| Patient No. | NIHSS1 | NIHSS2 | NIHSS3 | NIHSS4 | NIHSS5 |
| --- | --- | --- | --- | --- | --- |
| 1 | 1 | 1 | 0 | 0 | 0 |
| 2 | 2 | 1 | 1 | 0 | 0 |
| 3 | 2 | 1 | 0 | 0 | 0 |
| 4 | 2 | 1 | 1 | 0 | 0 |
| 5 | 1 | 1 | 1 | 1 | 1 |
| 6 | 2 | 2 | 1 | 1 | 1 |
| 7 | 2 | 1 | 1 | 0 | 0 |
| 8 | 1 | 0 | 0 | 0 | 0 |
| 9 | 4 | 3 | 1 | 0 | 0 |
| 10 | 3 | 1 | 0 | 0 | 0 |
| 11 | 4 | 3 | 1 | 1 | 1 |
| 12 | 4 | 3 | 1 | 0 | 0 |
| 13 | 5 | 3 | 1 | 1 | 1 |
| 14 | 8 | 4 | 2 | 2 | 1 |
| 15 | 8 | 3 | 2 | 2 | 1 |

Note: NIHSS 1-5 represent the speciﬁc NIHSS for each patient

at each time point.

**TABLE 2 | Demographic date of the NC group**

| NC patients No. | 1 | 2 | 3 | 4 | 5 | 6 | 7 | 8 | 9 | 10 | 11 | 12 | 13 | 14 | 15 |
| --- | --- | --- | --- | --- | --- | --- | --- | --- | --- | --- | --- | --- | --- | --- | --- |
| Age (year) | 56 | 54 | 54 | 48 | 65 | 52 | 53 | 70 | 52 | 61 | 54 | 54 | 63 | 45 | 57 |
| Gender | F | M | M | M | M | M | M | M | M | M | F | M | F | M | F |

NC, normal control

**Figure Legends**

**sFIGURE 1-7 | A.** Barplot depicting the mean (and standard error) GMV in the ROIs [in the CL uvula (sFigure 1), in the CL culmen (sFigure 2), CL, in the CL putamen (sFigure 3), in the IL tuber/tonsil (sFigure 4), in the IL middle frontal gyrus (sFigure 5), in the IL ventral anterior of thalamus (sFigure 6), in the IL mediodorsal thalamus (sFigure 7)] between the 2 groups across time. The asterisk indicates significantly differences at *p*＜0.05. **B.** Barplot depicting the results of the interaction effects in the ROIs [in the CL uvula (sFigure 1), in the CL culmen (sFigure 2), in the CL putamen (sFigure 3), in the IL tuber/tonsil (sFigure 4), in the IL middle frontal gyrus (sFigure 5), in the IL ventral anterior of thalamus (sFigure 6), in the IL mediodorsal thalamus (sFigure 7)]. The asterisk indicates signiﬁcant differences at *p*＜0.001 (uncorrected). CL = contralateral; IL = ipsilateral. NC = normal control; PI = pontine infarction. VA = ventral anterior; MD = mediodorsal.
